# Decision Support for Oropharyngeal Cancer Patients Based on Data-Driven Similarity Metrics for Medical Case Comparison

**DOI:** 10.3390/diagnostics12040999

**Published:** 2022-04-15

**Authors:** Julia Buyer, Alexander Oeser, Nora Grieb, Andreas Dietz, Thomas Neumuth, Matthaeus Stoehr

**Affiliations:** 1 Innovation Center Computer Assisted Surgery (ICCAS), 04103 Leipzig, Germany; nora.grieb@medizin.uni-leipzig.de (N.G.); thomas.neumuth@medizin.uni-leipzig.de (T.N.); 2 Head and Neck Surgery, Department of Otolaryngology, University Hospital Leipzig, 04103 Leipzig, Germany; andreas.dietz@medizin.uni-leipzig.de

**Keywords:** clinical decision support systems, case-based reasoning, similarity analysis, head and neck cancer, diagnostic patient model

## Abstract

Making complex medical decisions is becoming an increasingly challenging task due to the growing amount of available evidence to consider and the higher demand for personalized treatment and patient care. IT systems for the provision of clinical decision support (CDS) can provide sustainable relief if decisions are automatically evaluated and processed. In this paper, we propose an approach for quantifying similarity between new and previously recorded medical cases to enable significant knowledge transfer for reasoning tasks on a patient-level. Methodologically, 102 medical cases with oropharyngeal carcinoma were analyzed retrospectively. Based on independent disease characteristics, patient-specific data vectors including relevant information entities for primary and adjuvant treatment decisions were created. Utilizing the *ϕK* correlation coefficient as the methodological foundation of our approach, we were able to determine the predictive impact of each characteristic, thus enabling significant reduction of the feature space to allow for further analysis of the intra-variable distances between the respective feature states. The results revealed a significant feature-space reduction from initially 19 down to only 6 diagnostic variables (*ϕK* correlation coefficient ≥ 0.3, *ϕK* significance test ≥ 2.5) for the primary and 7 variables (from initially 14) for the adjuvant treatment setting. Further investigation on the resulting characteristics showed a non-linear behavior in relation to the corresponding distances on intra-variable level. Through the implementation of a 10-fold cross-validation procedure, we were further able to identify 8 (primary treatment) matching cases with an evaluation score of 1.0 and 9 (adjuvant treatment) matching cases with an evaluation score of 0.957 based on their shared treatment procedure as the endpoint for similarity definition. Based on those promising results, we conclude that our proposed method for using data-driven similarity measures for application in medical decision-making is able to offer valuable assistance for physicians. Furthermore, we consider our approach as universal in regard to other clinical use-cases, which would allow for an easy-to-implement adaptation for a range of further medical decision-making scenarios.

## 1. Introduction

According to the global cancer statistics (GLOBOCAN 2018) nearly 93,000 new cases of oropharyngeal squamous cell carcinoma (OPSCC) were reported worldwide in 2018 [1]. Lately, the incidence of OPSCC is significantly increasing in many countries worldwide, particularly due to positive human papillomavirus (HPV)-related OPSCC [2]. HPV, primarily type 16, is recognized as a risk factor and important prognostic factor alongside tobacco and alcohol consumption [3]. Nevertheless, the actual therapeutic decision for OPSCC is currently not differentiated according to HPV status. Instead, it is essentially based on the individual situation of the patient and his or her anatomical and biomedical conditions. While early-stage OPSCCs are usually treated by surgery or radiation therapy, more advanced stages require multimodal therapeutic concepts depending on the pathological indication. These may include invasive surgical procedures as well as adjuvant radiation or combined radiochemotherapy [4,5]. In cases of unresectable tumors, definitive radiochemotherapy is indicated. For recurrent or metastatic disease, new therapeutic options in the field of checkpoint immunotherapy have been approved. These represent a valuable addition to established conventional chemotherapies by blocking inhibitory immune checkpoint signaling pathways to reactivate immune response against cancer [6]. Activation of the PD-1 protein, which can be expressed by T cells, in response to PD-L1, leads to inhibition of the immunological response of T cells and serves as a mechanism to bypass the tumor immune system. Anti-PD-1/PD-L1 immune checkpoint inhibitors (ICIs) can inhibit suppressive signaling through the PD-1/PD-L1 pathway and enhance antitumor immune activity [7,8]. Due to individual tumor characteristics, differences in resectability and comorbidities that may conflict with radio- or even more chemotherapy, a personalized view of the diagnostic and therapeutic process becomes necessary. This includes adjusted diagnostics and individualized decision-making to provide optimal outcomes and a valuable quality of life for the individual patient.

To consider all personal patient-related factors, the evaluation of ideal treatment strategies for OPSCCs is currently being discussed in interdisciplinary tumor boards. In these meetings, specialists from different disciplines evaluate the available options in order to find the best possible therapy for a specific patient case. The following disciplines are usually represented: otorhinolaryngology, head and neck surgery, maxillofacial surgery, pathology, radiology, radiation therapy, as well as medical oncology [9]. Making such complex clinical decisions involves a set of individual considerations. The particular knowledge required to act in the patient’s favor comes from various sources of information such as learned expertise, specialist publications, and individual experience [10]. Verifiable results from significant medical studies or clinical trials are considered a level of safety as they represent the current state of clinical evidence [11]. This evidence also serves as a foundation for the preparation of clinical practice guidelines (CPG), which are provided by several medical associations. This overall process, also defined as evidence-based medicine (EBM), represents one current baseline for making medical decisions [12,13].

Although the concept of EBM integrates medical science and research, it provides general practice recommendations. So, it is therefore not an individual “instruction manual”, but must be applied to the individual patient according to the specific circumstances. Therefore, the clinical experience that a clinician accumulates during his or her professional career should not be underestimated in the diagnostic and decision-making process. Most judgments concerning specific criteria of the patient are made based on the clinician’s individual knowledge, training, and experience.

According to Lakoff et al., experience does not refer to memory, i.e., the result of interaction with the environment, but characterizes the immediate encounter, i.e., the process of repeated sensorimotor interaction with the environment in the sense of a repetitive action [14]. This progressively shapes and links the functional neuron groups involved in this process more effectively. Experience thus changes the neuronal connection patterns of the brain. This implies that the diagnosis and therapy finding of current patient cases are cognitively compared with similar patient and diagnostic profiles of the past. For very unusual, rare, and complex cases, for which even highly trained clinicians may lack the experience, this described process of decision-making reaches its limit and can no longer guarantee the optimal strategy for an individual patient [15]. Similarity analysis and comparison with previous cases could therefore form a valuable part of selecting an optimal diagnostic and therapeutic strategy. By means of an IT-supported process, it should be possible to access a broad knowledge base of patient cases. Based on the human cognitive process, an automatic search function can be used to evaluate specific diagnostic results of comparable patient cases and their courses for the current research question.

The idea of comparing a new problem with a similar previous situation found its beginnings in the 1980s and has been tried to establish since then [16]. As a cognitive similarity to clinical decision-making based on expertise coupled with the duality of subjective and objective knowledge, the term case-based reasoning (CBR) was introduced with the main principle: “similar problems have similar solutions” [16]. Considering the enormous potential of CBR for automated systems in clinics, the capability has yet to be achieved with suitable technologies, since other fields already utilize similar approaches. Similarity analysis is used in the medical context for DNS and protein analysis, for example, but is also used in many other domains [17]. It already forms an omnipresent and indispensable part in the context of recommendation systems. Based on the analysis of user behavior, suggestions for online shopping (“customers who bought this item also bought...”), music and movie streaming, or e-learning applications already influence decisions in our everyday life. To make recommendations, many member profiles with similar preferences and tastes are matched with the current user profile and the most suitable objects are recommended in a personalized catalog according to the collaborative technique of recommendation systems [18]. This already established concept should now also relieve medical staff in their everyday work.

The consideration of computational similarity analysis for patients is a well-known approach that has been thoroughly investigated throughout the years [19]. Especially since the advent of algorithmic analysis and machine learning (ML), methods such as k-nearest neighbors (kNN) and associated solutions have been applied to this problem with large success [20,21,22,23]. However, while those similarity metrics are well suited for the identification of similar (vector-based) abstractions of patients, they do only account for differences at a variable level (i.e., two patients with the same gender or almost equally distributed expressions in the blood count) but they do not consider the distances between individual variable states (i.e., between two categorical variables that are not equidistant regarding their influential factors, e.g., general performance status (ECOG) or other medical staging systems). While multiple measures that address this modality (also known as overlap measure) exist, they do only account for categorical variables. Since medical data sets are often subject to mixed variable types, solutions that are able to process those diverse entities are required.

To overcome those current methodological limitations in similarity search among patients, we present a novel approach that considers the intra-variable similarity of clinical cases based on mixed-type variables by using the *ϕK* correlation coefficient [24]. Due to this procedure, we also introduce a novel real-world application for the stated *ϕK* metric and evaluate its suitability for the task of patient matching. Accordingly, the main aim of the presented method is to contribute to comprehensive and objective (unbiased) assistance in case-based reasoning and thus also to the therapy decision process in the long term. In conclusion, this methodology made it possible to identify an objective selection of decisive diagnostic features and their individual impact on primary and adjuvant treatment decisions in the head and neck tumor board.

## 2. Materials and Methods

### 2.1. Information Modeling Creating a Patient-Specific Vector

**Figure 1:**
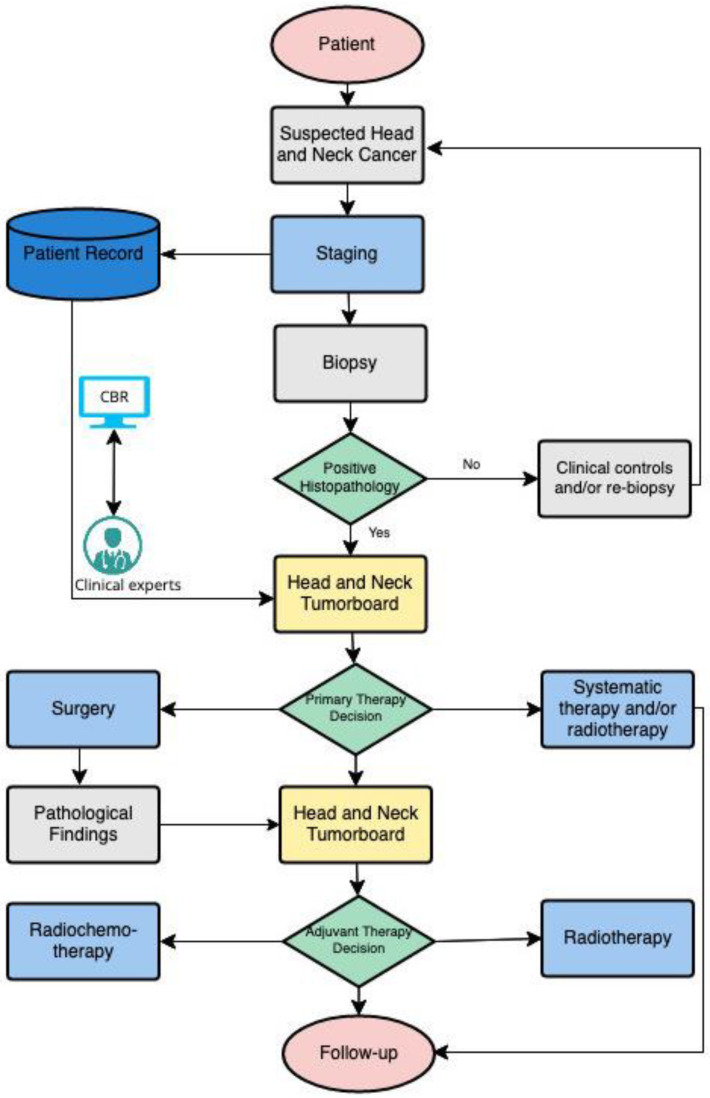
Process flow of primary and adjuvant diagnostic and therapy decision.

In order to adequately compare OPSCC patient cases, it is necessary to determine the context-specific variables (features) that are considered relevant to decision-making in relation to a corresponding endpoint (see Figure 1). In the present case, this endpoint relates to the primary and adjuvant treatment decision. Thus, relevant and specific characteristics were initially identified from the diagnostic results using the hospital’s internal clinical information system and then transferred into patient- and diagnosis-related features. For primary treatment, in the patient category, age, severe pre-existing conditions, and the ECOG score, a general performance measure, are decisive factors for diagnostic and therapeutic management (see exemplary patient data in supplement Table S1). While as diagnostic features, factors such as tumor size, infiltration of certain structures, possible metastases, as well as histo- and molecular-pathologic characteristics are important in assessing whether the tumor is resectable or chemotherapy is tolerable (see Table S2). Provided that surgical therapy is successfully evaluated in terms of achieving complete tumor resection with clear margins and optimal quality life expected postoperatively, potential adjuvant treatment is discussed in a postoperative tumor board based on the definitive pathologic findings. The histopathological report of the surgical resection should include tumor localization, tumor size, histological tumor type and grading, lymph vessel invasion, blood vessel invasion and perineural invasion, locally infiltrated structures, number and size of affected lymph nodes, presence of extracapsular extension, and the resection status (see Table S3). In addition, immunohistochemical scores such as the combined positive score (CPS) and the tumor proportion score (TPS) are also acquired to estimate PD-L1 expression. The CPS score evaluates the number of PD-L1 positive cells (tumor cells, lymphocytes, macrophages) relative to all viable tumor cells. TPS assesses the percentage of PD-L1 positive tumor cells in proportion to all viable tumor cells [25]. The result of this process is a patient-specific vector of independent information entities, which shows the related medical factors influencing therapy decisions in a structured format. In this context, Tables S1 and S2 each form the characteristic constellations for the primary decision scenario and Tables S1 and S3 for the adjuvant therapy.

To establish the dataset, 102 patient cases with OPSCC from the University Hospital of Leipzig were retrospectively analyzed. All of them were previously discussed by a team of interdisciplinary experts in the head and neck tumor board. We were able to only capture complete patient data without any missing information during this process.

### 2.2. Data-Driven Reduction of the Feature Space Using the PhiK Correlation Coefficient

Since not every feature is equally important in the context of making a therapeutic decision, a data-driven metric for expressing the individual weight of that information needed to be derived from the data set. To achieve this, we first split the data into a training (81 patients) and test set (21 patients) to enable later verification of our approach with previously unseen data (patient data that was not used to derive feature space reduction and intra-variable analysis). Based on the training set, we then calculated the individual correlation of each feature in relation to the recorded treatment decision using the PhiK (*ϕK*) package (version 0.9.12) in a Jupyter notebook python environment [24]. The *ϕK* coefficient is based on a refined version of Pearson’s χ^2^ contingency test to evaluate the independence of two or more variables through an algorithmic calculation without restrictions to a single variable type. Thus, it enables the parallel consideration of categorical, ordinal, and interval variables, which is a crucial characteristic when dealing with medical data that is usually represented in mixed-type data columns, e.g., age (ordinal), ECOG (categorical). In contrast to more traditional metrics, such as *Pearson’s r*, it also accounts for non-linear behavior between variables, which is another important characteristic regarding medical data including artificial scoring systems to express certain medical modalities (e.g., TNM-staging, ECOG). The PhiK package allows for the calculation of a correlation matrix using its own *ϕK* coefficient as the associated metric. While there is currently no gold standard regarding the correlation threshold, we defined scores greater than 0.3 to be significant for our analysis. The coefficient itself ranges from 0 to 1.

In a second step, we then evaluated the resulting features in terms of their statistical significance using the integrated PhiK significance based on a modified *p*-value calculation [24]. The algorithm then calculates a Z-value for each possible feature constellation which can then be obtained in a matrix-based representation according to the previous correlation matrix. For the performed analyses, we have determined a Z-value greater than 2.5 to be significant.

### 2.3. Analysis of Intra-Variable Behavior to Enable Granular Similarity Scoring

From a clinical point of view, there may be a difference in terms of treatment capacity whether a patient has ECOG = 1 or ECOG = 2, whereas no relevant distinction is usually made here between ECOG = 0 and ECOG = 1 states. Therefore, we further refined our analysis to account for intra-variable behavior in the remaining features (after reduction) with the goal to quantify the individual differences between the respective variable states. We therefore performed the same *ϕK*-based correlation and significance tests in relation to the therapy target variable while limiting the respective input feature states to every possible pairwise permutation schema, e.g., ECOG 0/ECOG 1, ECOG 0/ECOG 2, and ECOG 1/ECOG 2. In this way, we were able to calculate the numeric differences between the resulting clusters, allowing us to derive the amount of similarity or distance that results from looking at the individual states rather than the overall feature.

### 2.4. Consolidation of the Findings into a Similarity Metric

Based on the inter- and intra-variable analysis of the considered features, we were able to construct a weight-matrix that integrates the *ϕK* correlation coefficients for all possible state permutations. Based on this, we suggest the implementation of the derived findings as additional factors to the calculation of similarity in the following way:

$$Similarity\;\left(S\right)=1-\frac{\sum_{i=1}^{n}w_{i}}{n}$$

Thereby, n represents the number of features in a patient vector that is considered for similarity analyses with a range of other same-type vectors in an iterative way. The weight factor w represents the associated values from the weight-matrix that account for the respective correlation for each constellation of variable states. Due to w, a relatively small *ϕK* correlation coefficient also results in a small distance as it has been shown that the derivation between both factors is of less importance for the respective decision scenario. Consequently, if the normalized sum of all feature correlation is small, it follows that the distance between two patient vectors is small, which then results in a high similarity value S.

### 2.5. Evaluation of the Approach

To verify our approach, we further implemented an initial evaluation process by performing similarity searches among the test set (new and unseen patient data) with the training set. In this scenario, we considered a difference in variable states as relevant, if the calculated weight surpassed a score of 0.5. All other constellations were thus considered to be similar. If one or more matches (defined as patients to be the same or similar in all considered features) for a case in the test set were found according to this procedure, we then checked if their corresponding therapy selection was equal among all findings. For example, if our approach identified cases B and C as two similarity matches for case A, and all cases were treated equally, this would result in a perfect evaluation score of 1.0. If differences were found in the recorded treatments, the score would decrease accordingly. Finally, we have calculated a final evaluation score through summing up the individual results and dividing them by the sum of all found matches. To account for unrepresentative effects caused by only a one-time random selection of cases in the train-test-split, we implemented a 10-fold cross validation with randomly assigned cases to the respective test (*n* = 21) and training (*n* = 81) cohorts during each fold. The overall evaluation metric (evaluation score) is thus defined as the mean of the individual per-run outcomes.

## 3. Results

### 3.1. Statistical Description of the Data Set

With an average age of 60.4 years and a male share of 74.5% (76 patients), the data set represents typical patients with OPSCC. A total of 25.5% (26 patients) of the documented patients are female. This corresponds to a female to male ratio of 1:2.9. Overall, 50% (51 patients) had ECOG status 0, which means normal unrestricted activity as before the disease. Whereas 41.2% (42 patients) already have minor physical limitations, which is encoded by an ECOG status of 1. The remaining have further restrictions and an ECOG status of 2, which means that the therapy options for invasive procedures may be limited (see Table 1).

At the time of diagnosis, 81.4% (83 patients) of the cases already had affected lymph nodes and 59.8% (61 patients) had a tumor size of more than 4 cm (see Table 2). In 8.8% (9 patients), distant metastases were already detectable at time of diagnosis. Remarkable are the tumor infiltrations into neighboring structures, such as in 58.8% (60 patients) into the tongue musculature, in 8.8% (9 patients) into the nasopharynx, and in 23.5% (24 patients) into the hypopharynx. In particular, the involvement of non-lymphatic structures, including the internal jugular vein (IJV), spinal accessory nerve (SAN), and sternocleidomastoid muscle (SCM) determine the surgical management of the neck in OPSCC [26]. The frequencies for these are distributed as follows in our dataset: IJV: 18.6% (19 patients), SAN: 5.9% (6 patients), and SCM 13.7% (14 patients). 

Risk factors as well as possible indicators for adjuvant treatment are included in the final histopathological findings (see Table 3). For instance, in our dataset, extracapsular spread of the lymph node metastasis was observed in 37.3% (38 patients). Positive resection margins were detected in 7.8% (8 patients). Perineural and lymphatic invasion were found both in 76.5% (78 patients) of the pathological examinations, vascular invasion in 6.9% (11 patients).

Nevertheless, 66.7% (68 patients) of the cases were treated primarily surgically with a complete, so-called R0 resection rate of 88.2% (60 patients). Of those who underwent surgery, an indication for postoperative adjuvant treatment was assessed in 91.2% (62 patients), dividing into 58.1% (36 patients) with adjuvant radiochemotherapy and 41.9% (26 patients) with adjuvant radiotherapy alone. A total of 27.5% of cases (28 patients) were treated with definitive radiochemotherapy. The remaining 5.9% (6 patients) received best supportive care, which is not a curative approach, but inherits the main aim to relieve the symptoms and achieve the best possible quality of life (see Table 4).

### 3.2. Identification of Diagnostic Factors for the Primary Treatment Scenario

**Figure 2:**
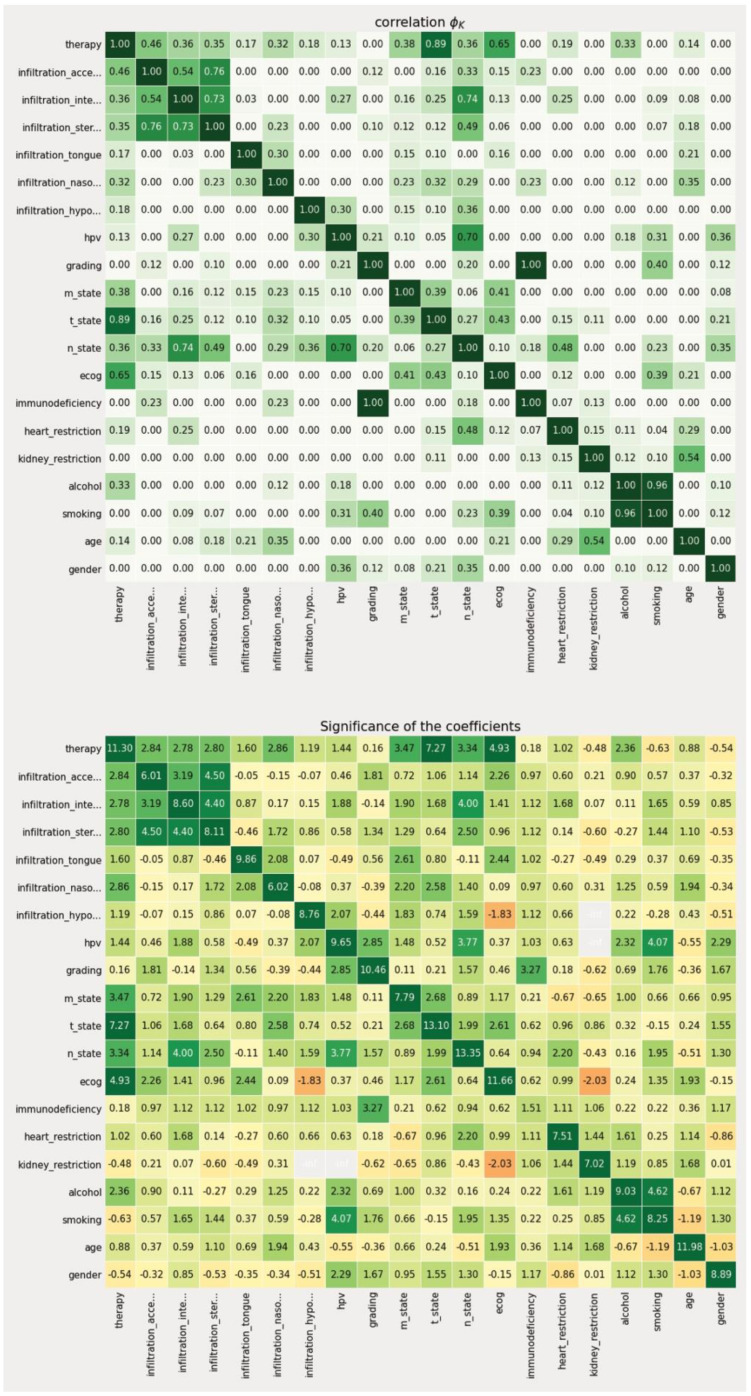
Correlation and significance matrix of the *ϕK*-based analysis of the primary therapy decision scenario.

Regarding the primary treatment scenario, the utilization of *ϕK*-based correlation and significance analysis identified six diagnostic factors with a representative correlation coefficient above 0.3 and a Z-value above 2.5 (see Figure 2). Those included T-state (correlation: 0.38, significance: 7.27), N-state (correlation: 0.36, significance: 3.34), M-state (correlation: 0.89, significance: 3.47), ECOG (correlation: 0.65, significance: 4.93), as well as the infiltrations of the sternocleidomastoid muscle (correlation: 0.35, significance: 2.80), the internal jugular vein (correlation: 0.36, significance: 2.78), the nasopharynx (correlation: 0.32, significance: 2.86), and the accessory nerve (correlation: 0.46, significance: 2.84). From a medical point of view, this corresponds to the clinical factors most weighted in the tumor board. For primary therapy, tumor size and infiltration of certain structures play a decisive role in the diagnostic process, as this influences resectability. The ECOG concludes a clinical assessment of a patient’s general performance and therefore, correlates with the tolerability of invasive procedures such as surgery, radiotherapy, and even more chemotherapy.

### 3.3. Analysis of Intra-Variable Distances

**Figure 3:**
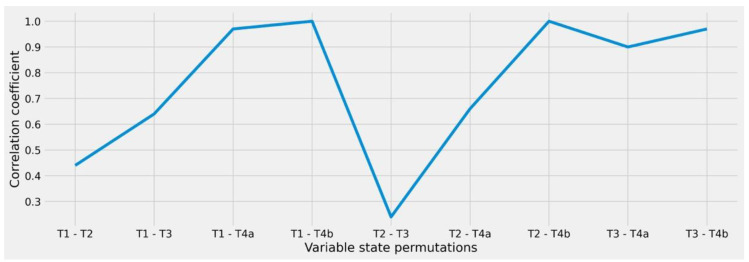
Correlation coefficient of the T-state variable under different state permutations. The T-state (tumor state) is defined as a multi-factorial metric that classifies a range of tumor characteristics, e.g., size or an infiltration of specific anatomic regions.

Based on the fact that the M-state as well as the identified infiltrations are represented as binary states that can either be present or not present (0 or 1 for the M-state, respectively), those factors did not account for intra-variable investigation. Thus, the overall correlation of the feature can be further considered. In terms of the T-state, our analysis showed a non-linear behavior which closely adapts to clinical expectations (see Figure 3). Consequently, extreme differences in staging (i.e., T1 to T4) do also have extreme deviations while smaller distances do have smaller impact on the therapeutic decision and thus, more similarity during case comparison.

**Figure 4:**
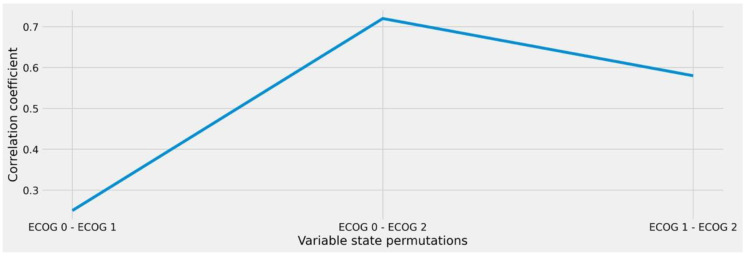
Correlation coefficient of the ECOG variable under different state permutations. The ECOG status is a medical classification system to express the activity index and overall fitness of an individual patient.

In a similar way, the analysis of ECOG provided equally comprehensible results that clearly show the value of considering intra-variable distances to derive medical case similarity (see Figure 4). While the derivation of ECOG 0 and ECOG 1 showed almost no impact on assessing two individuals as different during therapy decision-making, larger distances (i.e., ECOG 0 and ECOG 2) carry tremendous differences. This behavior would have not been obvious from considering the overall feature correlation of 0.65 (see Figure 2) during similarity calculation.

Implementing the previously introduced 10-fold cross evaluation approach for similarity-based case matching through unseen test data, we were able to identify a median of eight cases from the testing cohort with one or more identified matches from the training cohort. Based on the fact that all those identified matches shared an equal treatment modality with the corresponding test case, we were able to achieve a perfect evaluation score of 1.0.

### 3.4. Identification of Diagnostic Factors for the Adjuvant Treatment Scenario

In clinical practice, the decision to conduct postoperative (adjuvant) therapy is based on CPG, such as the NCCN guidelines, which specify exactly which characteristics require adjuvant therapy and if so, which particular strategies [27]. Thus, for example, a patient who has undergone a complete R0 resection with sufficient margins after surgery, along with a N0 status, would not require adjuvant therapy in many cases. However, in clinical practice, patients are still offered the option of adjuvant therapy, for example when certain risk factors such as expended tumor size (T3 and larger) or lymphatic (L1), venous (V1), or perineural invasion (Pn1) are identified.

**Figure 5:**
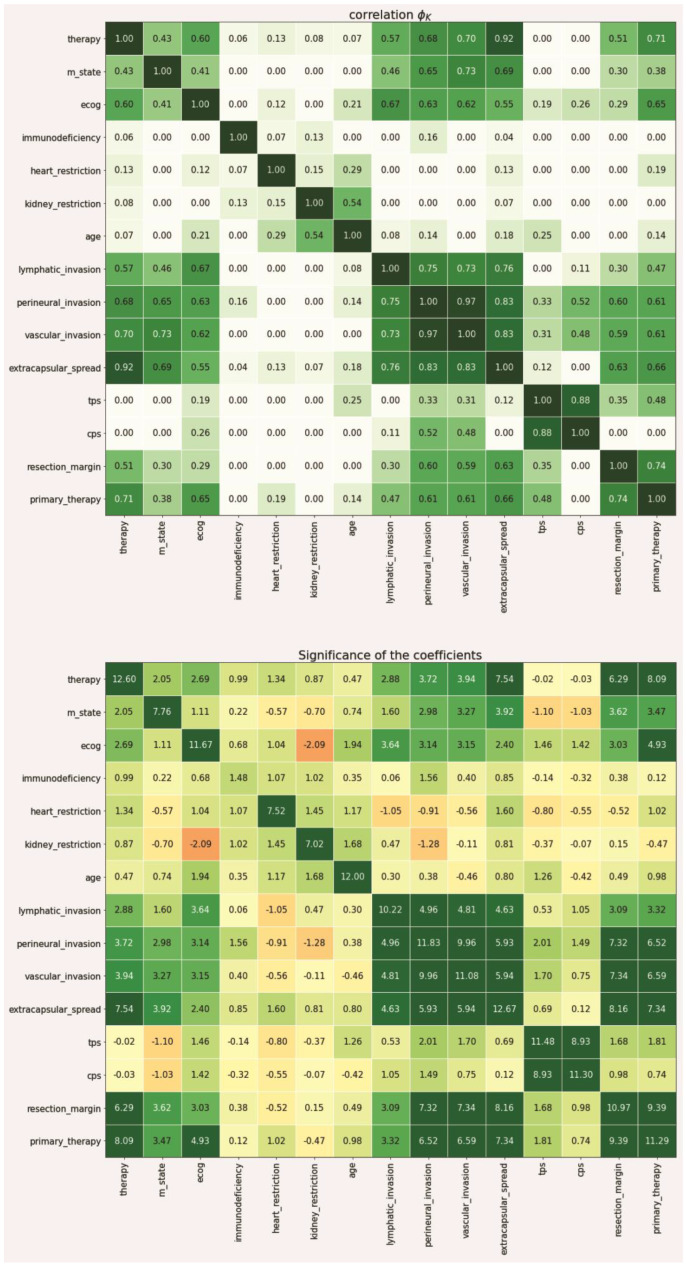
Correlation and significance matrix of the *ϕK*-based analysis of the adjuvant therapy decision scenario.

Our analysis identified seven diagnostic factors as relevant for adjuvant therapy decision (see Figure 5). Those included primary therapy (correlation: 0.71, significance: 8.09), ECOG status (correlation: 0.60, significance: 2.69), lymphatic invasion (correlation: 0.57, significance: 2.88), perineural invasion (correlation: 0.68, significance: 3.72), vascular invasion (correlation: 0.70, significance: 3.94), extracapsular spread (correlation: 0.92, significance: 7.54), as well as resection margin (correlation: 0.51, significance: 6.29). The factors identified by the application are also consistent with the clinical approach. The interdisciplinary tumor board for post-surgery treatment also considers various characteristics that determine the adjuvant therapy. Firstly, results from pathological diagnostics, such as a positive resection margin or extracapsular spread of the lymph node metastasis, are an indication for adjuvant therapy.

**Figure 6:**
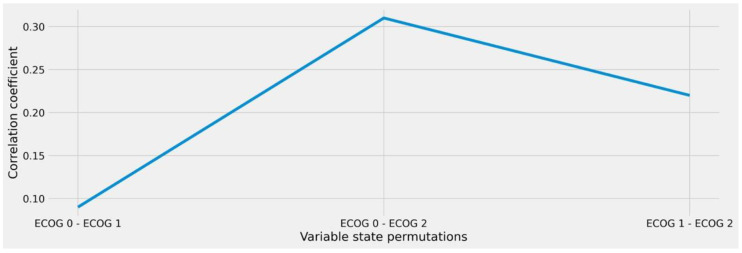
Correlation coefficient of the ECOG status variable under different state permutations for the adjuvant therapy decision scenario.

While the presence of invasion in the distinct anatomical structures is again represented as binary expressions, we further investigated the ECOG status variable according to our presented approach. From the numbers (see Figure 6), we again perceive a non-linear behavior, which ranges between a correlation score of 0.06 and 0.32. For our approach, however, this relates to the fact that changes throughout those states need not to be considered during similarity matching: a fact which would have been the case if state deviations in the variable would have been considered in general (correlation: 0.60, see Figure 5). Further analysis would have also been necessary regarding the resection margin variable based on our stated method. However, since the presence of state R2 was only found once in the data, it was not possible to find matches in the train-test split. Thus, we considered it at an overall variable level (correlation: 0.51) during evaluation and only considered absolute matches throughout the respective states to be similar.

Based on the 10-fold cross evaluation procedure, we were able to identify a median of nine cases from the testing cohort with one or more matches from the training cohort. The results revealed a mean evaluation score of 0.957 (minimum: 0.71, maximum: 0.96), which relates to a very high accuracy in the identification of patients that received equal adjuvant therapeutic procedures.

**Figure A1:**
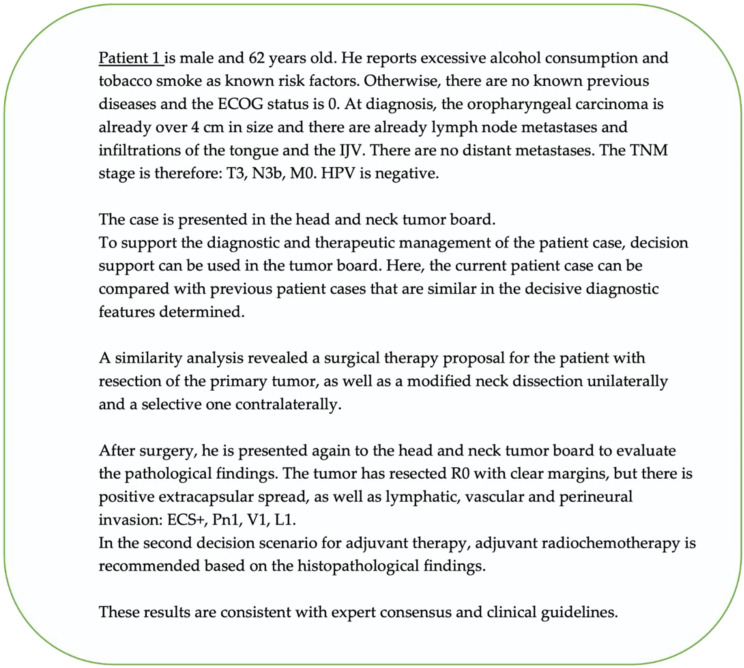
Clinical Use-Case illustrating how Medical Case Comparison could be used for Decision Support in a clinical setting.

For practical application of the model to support the diagnostic and therapeutic process, the model emphasizes a detailed determination of extracapsular extension of lymph nodes. For a patient with pT2 pN1 M0 (according to TNM classification 2017 [28]) OPSCC and HPV-16 positivity, there are critical differences in treatment decision-making. In particular, the absence of extracapsular extension has to be considered, which practically indicates radiotherapeutic adjuvance alone, whereas the presence of extracapsular extension requires combined adjuvant chemoradiation. Another example points out the significance of the ECOG status. In a patient with pulmonary metastasis from an OPSCC, the model refers to the evaluation of ECOG status to estimate chemo tolerability: A patient with ECOG 1 is likely to tolerate systemic chemotherapy, whereas a similar patient with ECOG 3 will probably not tolerate conventional chemotherapy. Another finding directs to the diagnostic assessment of nasopharyngeal infiltration of OPSCC regarding treatment decision, as indicated in Figure 5. How case comparison could be used in a clinical setting is also shown in Figure A1 in Appendix A.

## 4. Discussion

Based on our approach, an initial objective selection of crucial diagnostic features and their individual impact regarding primary and adjuvant therapy decisions in the head and neck tumor board could be established. Nevertheless, it should be noted that the determination of the introduced metrics is highly dependent on the underlying database. It must therefore be assumed that the results of our retrospective analysis of 102 patient cases are limited to some extent and would be more significant with the integration of more or other data sets. This research therefore serves as a proof-of-concept study. The outcomes presented in this paper should be considered as a starting point that needs to be further analyzed and verified by including additional case data. The precision of the decision is then proportional to the amount of case evidence provided. However, based on the trends and effects revealed by the utilized algorithms, we were able to agree with the results from the perspective of clinical professionals in the weighting of diagnosis-related factors. This indicates that the presented approach is likely to adapt to causal implications in the real-world setting (e.g., lowering the need for adjuvant treatment when an R0 resection with clear margins was achieved). 

In this work, we exclusively focused on the utilization of the *ϕK*-correlation coefficient to perform feature-space reduction and similarity scoring. While this method was mainly based on the fact that the integrated data set inherited a mixed-type variable constellation, the resulting sets for both treatment decision scenarios were purely categorical. This would have allowed for benchmarking our approach with other methodological solutions that are also capable of considering state differences among variables (i.e., Goodall measure or probability-based methods). However, since the main goal of our work was to present a novel solution to the problem of case-based reasoning, an in-depth comparison of our approach to other potential solutions was out of scope but should be considered in future works, also using further data sets to evaluate the generalizability. While this might go along with different outcomes regarding the resulting feature set (e.g., by integrating numerical variables such as laboratory measures), the issue of unprocessable complexity in the analysis of state permutations among two variables would require pre-processing, e.g., by transforming the respective values to z-scores. 

The analysis performed in this study considers only patients who were assessed and treated in a single hospital. Thus, the aspect of institutional bias cannot be completely dismissed. However, due to the generalized description and design of the methodological process, a simple transferability to a multicenter application is feasible. This could not only lead to a minimization of bias but could also make the process of identifying similar patient cases even more useful by extending the associated search area accordingly. However, this implementation is associated with a correspondingly high organizational and technical effort in practice as it would require the provision of a central repository for the structured input and storage of medical case data. It would also be necessary to ensure terminological consistency. This standardization also applies to the initial evaluation of the individual information entities for prior classification. 

Furthermore, it should be noted that for certain patient cases, there may be more than one possible treatment option and that the patient’s will should not be disregarded. This may lead to deviations between the tumor board decision and the intervention that is performed and documented in the electronic health record. Consequently, it is possible that patients who might be identified as similar by our approach might have received another treatment option than the one initially suggested to the patient. In a future setting that integrates our approach towards similarity calculation for case-based reasoning, this very likely circumstance should be addressed.

Although the methodological approach was presented and evaluated using the example of OPSCC, it requires very little adaptation for further use cases in both oncological and non-oncological contexts. For this purpose, the presented processes only need to be mapped to the respective domain and the results need to be interpreted and evaluated accordingly. 

This method may also be suitable for very rare and complex cases, where decision-making is further complicated when the available information and experience is limited. Therefore, misdiagnosis and incorrect treatment are more likely in rare and complex diseases due to insufficient knowledge and awareness [29]. A concise identification of objective, decisive diagnostic features and an analysis of similarity to previous cases can answer individual questions with the aim of determining the best possible diagnosis and treatment strategy for the patient. This adds quality and granularity to the decision-making process and potentially improves patient outcomes. In addition, the analyses provided may contribute to the training and expertise of health professionals. Particularly, beginners may benefit from this, which also enhances objectivity and quality control in hospital diagnostic and treatment processes.

While the provided solution is intended to offer rational and intuitive assistance in clinical decision-making, it still needs to be considered that medical cases provide enormous diversity and should not be exclusively evaluated by a set of features. However, our primary aim is to provide proper assistance in identifying relevant cases as a further source of evidence in the therapy decision process and not the specification of the decision itself.

## 5. Conclusions

In this paper, we developed and evaluated a novel approach to provide data-driven similarity analysis for medical cases to support the diagnosis and treatment process in clinical practice. By calculating the individual *ϕK*- correlation of each diagnostic feature in relation to the registered treatment decision and evaluating its significance, it was possible to identify both patient- and diagnosis-related factors that are consistent with the clinical assessment of experts and the clinical practice guidelines. Based on the implemented procedure, we were able to evaluate a novel real-world application that can benefit from the theoretical works by Baak et al. [24] and the resulting *ϕK* correlation coefficient in a meaningful way.

This allows an individualized diagnostic assessment of the patient, potentially reducing the patient’s waiting time for treatment proposals and enabling the application of the most effective treatment method. Since the individual expertise of the collaborative members of a tumor board highly depends on the individual participants, the presented method can introduce a new layer of competence by enabling case comparison. This helps to tackle uncertainty or decision bias, thus providing sufficient support to the diagnosis and treatment process in order to improve patient outcomes. 

## Figures and Tables

**Table 1:** Statistical summary of the patient-related factors for primary and adjuvant treatment decision.

Patient-Related Features N = 102		Absolute Frequency	Relative Frequency
Gender	Male	76	0.745
	Female	26	0.255
Consumption	Alcohol	81	0.794
	Tobacco Smoke	87	0.853
ECOG Status	ECOG 0	51	0.5
	ECOG 1	42	0.412
	ECOG 2	9	0.088
	ECOG 3	0	0
Pre-existing condition	Heart Restriction	13	0.127
	Kidney Restriction	11	0.108
	Immunodeficiency	1	0.010

**Table 2:** Statistical summary of the diagnosis-related factors for primary treatment decision.

Diagnosis-Related Features N = 102		Absolute Frequency	Relative Frequency
T State	Tx	0	0
	T1	9	0.088
	T2	32	0.314
	T3	27	0.265
	T4a	29	0.284
	T4b	5	0.049
N State	Nx	0	0
	N0	19	0.186
	N1	19	0.186
	N2	40	0.392
	N3	24	0.235
M State	Mx	2	0.020
	M0	91	0.902
	M1	9	0.088
HPV status	positive	38	0.373
	negative	64	0.627
Grading	G1	1	0.010
	G2	60	0.588
	G3	41	0.402
Infiltration	Nasopharynx	9	0.088
	Hypopharynx	24	0.235
	Tongue	60	0.588
	Internal jugular vein	19	0.186
	Spinal Accessory Nerve	6	0.059
	Sternocleidomastoid Muscle	14	0.137

**Table 3:** Statistical summary of the diagnosis-related factors for adjuvant treatment decision.

Diagnosis-Related Features N = 102		Absolute Frequency	Relative Frequency
Resection Margin	No surgery	34	0.333
	R0	60	0.588
	R1	7	0.069
	R2	1	0.010
Extracapsular Spread	Positive	38	0.373
	Negative	32	0.314
	Not measurable	32	0.314
Vascular Invasion	Vx	34	0.333
	V0	57	0.598
	V1	11	0.069
Perineural Invasion	Pnx	12	0.118
	Pn0	12	0.118
	Pn1	78	0.765
Lymphatic Invasion	Lx	12	0.118
	L0	12	0.118
	L1	78	0.765

**Table 4:** Statistical summary of the treatment-related factors for primary and adjuvant treatment decision.

Treatment-Related Features N = 102		Absolute Frequency	Relative Frequency
Primary treatment	Surgery	2	0.020
	Surgery + Selective neck dissections	57	0.559
	Surgery + Modified neck dissection unilateral, Selective neck dissection contralateral	7	0.069
	Surgery + Radical neck dissection unilateral, Selective neck dissection contralateral	2	0.020
	Definitive radiochemotherapy	28	0.275
	Best supportive care	6	0.059
Adjuvant treatment	None	40	0.353
	radiotherapy	26	0.255
	radiochemotherapy	36	0.392

## Data Availability

Not applicable.

## Supplementary Materials

The following supporting information can be downloaded at: https://www.mdpi.com/article/10.3390/diagnostics12040999/s1, Table S1: Patient-related factors for the primary treatment decision of three example patients of the dataset. Table S2: Diagnosis-related factors for the primary treatment decision of three example patients of the dataset. Table S3: Diagnosis-related factors for the adjuvant treatment decision of three example patients of the dataset.
