# Supplementary material for: Decision Support for Oropharyngeal Cancer Patients Based on Data-Driven Similarity Metrics for Medical Case Comparison
Source: Diagnostics (Basel). 2022 Apr 15;12(4):999. doi: 10.3390/diagnostics12040999 (PMC9029638; doi:10.3390/diagnostics12040999)
Supplement: Supplementary file 1 [file diagnostics-12-00999-s001.zip › diagnostics-1652528-supplementary.pdf]

Supplement Tables

Table S1: Patient-related factors for the primary treatment decision of three example patients of the dataset.

Patient	Gender	Age	ECOG	Pre-existing illness	Alcohol Consumption	Tobacco Smoke
1	Male	62	0	None	Yes	Yes
2	Female	59	1	None	Yes	Yes
3	Male	70	2	None	Yes	Yes

Table S2: Diagnosis-related factors for the primary treatment decision of three example patients of the dataset.

Patient	T-State	N-State	M-State	HPV	Grading	Infiltrations
1	T3	N3b	M0	Negative	G2	Tongue, IJV
2	T4a	N2c	M0	Positive	G2	Tongue, Hypopharynx, Nasopharynx
3	T4b	N3	M1	Negative	G2	Tongue, IJV

Table S3: Diagnosis-related factors for the adjuvant treatment decision of three example patients of the dataset.

Patient	Primary therapy	Resection margin	Vascular invaion	Perineural invasion	Lymphatic invasion	ESC
1	Surgery	R0	V1	Pn1	L1	Positive
2	Definitve radiochemo-therapy	Not applicable	Vx	Pnx	L1	Negative
3	Palliative radiotherapy	Not applicable	Vx	Pnx	Lx	Negative
